# Medicina de Precisão: A Tomografia por Emissão de Pósitrons com 18F-FDG pode Identificar Fenótipos de Cardiotoxicidade?

**DOI:** 10.36660/abc.20220393

**Published:** 2022-07-07

**Authors:** Claudio Tinoco Mesquita, Maria Fernanda Rezende

**Affiliations:** 1 Universidade Federal Fluminense Niterói RJ Brasil Universidade Federal Fluminense, Niterói, RJ – Brasil; 2 Hospital Pró-Cardíaco Rio de Janeiro RJ Brasil Hospital Pró-Cardíaco, Rio de Janeiro, RJ – Brasil; 3 Hospital Vitória e Samaritano da Barra Rio de Janeiro RJ Brasil Hospital Vitória e Samaritano da Barra, Rio de Janeiro, RJ – Brasil

**Keywords:** Cintilografia, Tomografia por Emissão de Pósitrons, Toxicidade

A publicação nos Arquivos Brasileiros de Cardiologia do artigo de Dourado et al.^1^ deve ser vista com bastante interesse por cardiologistas que buscam a Medicina de Precisão. Neste estudo, os autores, em setenta pacientes com linfoma, a intensidade de captação de 2-[18F]-fluoro-2-desoxi-D-glicose (18F-FDG) pelo miocárdio por tomografia por emissão de pósitrons associada a tomografia computadorizada (PET/CT) antes, durante e após quimioterapia. Os autores observaram progressivo aumento do metabolismo de glicose no ventrículo esquerdo do PET/CT basal para o PET/CT intermediário, e desse para o PET/CT pós-terapia. Mais da metade dos pacientes analisados demonstraram um aumento ≥ 30% na intensidade de captação de glicose, conforme medida pelo SUV máxima no ventrículo esquerdo. Os autores inferem que o PET/CT é capaz de avaliar de modo confiável a intensidade de captação de 18F-FDG em pacientes com linfoma durante e após quimioterapia. Mais do que isto, os autores conseguiram identificar um grupo de pacientes em que a quimioterapia causou maior repercussão metabólica no ventrículo esquerdo.^1^ Esses achados podem contribuir para uma estratégia de identificação precoce de pacientes com maior sensibilidade à toxicidade cardíaca das drogas empregadas e de definição, de modo mais personalizada, de medidas de prevenção dos danos irreversíveis ao coração.

A Medicina de Precisão é comumente definida como uma abordagem para tratamento e prevenção de doenças que leva em consideração a variabilidade individual e a manifestação da doença em cada indivíduo. Para que isso ocorra de modo adequado, é necessário que se identifiquem mecanismos específicos de desenvolvimento das doenças e pontos chaves para implementação de abordagens eficazes.^2^ Esse processo, é conhecido como fenotipagem profunda, onde se identificam fenótipos subjacentes (endótipos) ao fenótipo comum inicial, permitindo um melhor direcionamento de abordagens terapêuticas.^3^

O 18F-FDG é uma sonda molecular sensível, capaz de avaliar tanto a expressão aumentada da captação de glicose em células tumorais viáveis como monitorar a efetividade da resposta terapêutica ao tratamento do câncer. Borde et al.^4^ foram um dos primeiros a demonstrar o impacto da toxicidade das antraciclinas na captação de FDG em um grupo de pacientes com aumento considerável da concentração do traçador após o tratamento. Os autores especularam que a dose de adriamicina administrada tenha atingido o limite individual e levou à ativação da via NRG-erb com aumento da utilização de glicose pelos miócitos. Estudos experimentais com uso de radioterapia na área cardíaca mostraram que a alta captação de FDG em um campo irradiado parece estar associada ao dano na microcirculação relacionada à lesão mitocondrial.^5^

**Figura 1:**
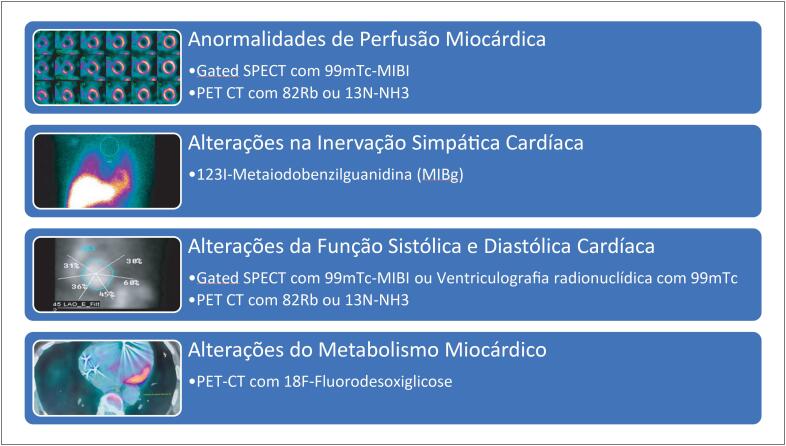
Principais aplicações da Medicina Nuclear na Detecção e Acompanhamento da Toxicidade Cardíaca do Tratamento do Câncer. MIBI: sestamibi; Rb: Rubídio; PET-CT: Tomografia por emissão de pósitrons acoplada com tomografia computadorizada; SPECT: Tomografia computadorizada de emissão de fóton único; NH3: amônia; Tc: Tecnécio.

No recente Posicionamento Brasileiro sobre o Uso da Multimodalidade de Imagens na Cardio-Oncologia da Sociedade Brasileira de Cardiologia,^6^ a técnica do PET-CT com 18F-FDG é mencionada no diagnóstico da cardiotoxicidade induzida pelos inibidores dos checkpoints imunológicos, visto que permite detectar e avaliar a extensão e até mesmo quantificar o processo inflamatório de diversas afecções cardiovasculares, tais como miocardite, pericardite e vasculites.^6^ Além do PET CT com 18F-FDG, existem muitas outras aplicações da medicina nuclear na avaliação da toxicidade do câncer ao coração. Na figura 1 podemos observar que a lista de aplicações da medicina nuclear está progressivamente aumentando, incluindo não somente exames para avaliação da função sistólica e diastólica ventricular (que só ficam anormais em fases mais avançadas do dano cardíaco), como também exames que avaliam processos mais sensíveis do coração como a perfusão, a inervação e o metabolismo da célula cardíaca.

Em suma, a medicina de precisão é um caminho de grande importância para a medicina atual. Como no exemplo do estudo de Dourado et al.,^1^ em que se encontrou um perfil molecular de resposta ao tratamento quimioterápico para um grupo de pacientes, acreditamos que também será individualizada, futuramente, a abordagem preventiva e terapêutica na cardio-oncologia. Alguns estudos experimentais têm sugerido que abordagens não-farmacológicas, como atividade física regular, podem ser úteis na prevenção da cardiotoxicidade induzida por quimioterapia, e que poderiam ser orientadas de modo mais preciso com a correta identificação dos pacientes em maior risco.^7^ Desta forma, a estratificação de pacientes e a compreensão das suas respostas celulares e bioquímicas aos diversos tratamentos permitirá uma abordagem personalizada, reduzindo a morbidade e aumentando as chances de bons desfechos do tratamento.
