# Supplementary material for: Medicina de Precisão: A Tomografia por Emissão de Pósitrons com 18F-FDG pode Identificar Fenótipos de Cardiotoxicidade?
Source: Arq Bras Cardiol. 2022 Jul 7;119(1):109–10. [Article in Portuguese] doi: 10.36660/abc.20220393 (PMC9352130; doi:10.36660/abc.20220393)
Supplement: Translation

# Translation: Precision Medicine: Can 18F-FDG PET Detect Cardiotoxicity Phenotypes?

**Authors:** Claudio Tinoco Mesquita, Maria Fernanda Rezende

**Affiliations:** 1 Niterói RJ Brazil Universidade Federal Fluminense, Niterói, RJ – Brazil; 2 Rio de Janeiro RJ Brazil Hospital Pró-Cardíaco, Rio de Janeiro, RJ – Brazil; 3 Rio de Janeiro RJ Brazil Hospital Vitória e Samaritano da Barra, Rio de Janeiro, RJ – Brazil

**Keywords:** Radionuclide Imaging, Positron-Emission Tomography, Toxicity

The publication of the article by Dourado et al.^1^ in the *Arquivos Brasileiros de Cardiologia* should be carefully considered by cardiologists engaged in Precision Medicine. In this study,^1^ the authors investigated the intensity of 2-deoxy-2[18F] fluoro-D-glucose (18F-FDG) myocardial uptake in 70 patients with lymphoma by positron emission tomography associated with computed tomography (PET/CT) scans before, during and after chemotherapy. They observed a progressive increase in glucose metabolism in the left ventricle from baseline PET/CT to interim PET/CT, and from interim PET/CT to post-therapy PET/CT. More than half of patients showed an increase of ≥ 30% in cardiac 18F-FDG uptake measured by left ventricular SUV max. The authors inferred that PET/CT is a reliable method to assess the intensity of 18F-FDG uptake in patients with lymphoma during and after chemotherapy. More importantly, the authors could identify a group of patients in which the metabolic effects of chemotherapy on the left ventricle were the greatest.^1^ These findings can contribute to a strategy for the early identification of patients who are more sensitive to cardiotoxicity of chemotherapeutic agents and for the definition of individualized preventive measures of irreversible myocardial damage.

Precision Medicine is commonly defined as an approach for disease treatment and prevention that takes into account individual variability and disease manifestation for each person. For this purpose, it is fundamental to clarify specific mechanisms of disease and key points for the implementation of effective interventions.^2^ This process is known as deep phenotyping, where endotypes, *i.e*., phenotypes underlying the common phenotype are identified, allowing a more effective guidance of therapeutic approaches.^3^

18F-FDG is a sensitive molecular probe that can evaluate not only an increased expression of glucose uptake by viable tumor cells but also monitor the effectiveness of therapeutic response to cancer treatment. Borde et al.^4^ were one of the first to demonstrate the impact of anthracycline toxicity on FGD uptake in a group of patients who showed a considerable increase in the tracer uptake after treatment. The authors speculated that the dose of adriamycin may have reached the individual limit, leading to activation of the NRG-erbB pathway and increased glucose utilization by the myocytes. Experimental studies with cardiac radiotherapy have shown that the increased FDG uptake in an irradiated field may be associated with microvascular damage related to the direct injury of the mitochondria by radiation.^5^

In the recent Brazilian position statement on the use of multimodality imaging in cardio-oncology,^6^ 18F-FDG PET/CT is mentioned in the diagnosis of cardiotoxicity induced by immune checkpoint inhibitors, as the method allows to detect, assess and even quantify the extent of inflammation in several cardiovascular disorders, including myocarditis, pericarditis and vasculitis.^6^ In addition to 18F-FDG PET/CT, other nuclear medicine techniques can be used to assess cardiac toxicity induced by cancer. Figure 1 illustrates that the list of the nuclear medicine applications has been progressively increasing, including not only tests for evaluation of systolic and diastolic functions (which become abnormal in advanced stages of cardiac damage only), but also tests that evaluate more sensitive processes in the heart, such as perfusion, innervation, and cell metabolism.

**Figure 1:** Main applications of nuclear medicine for detection and monitoring of cardiotoxicity in cancer treatment. MIBI: sestamibi; Rb: Rubidium; PET CT: positron emission tomography-computed tomography; SPECT: single-photon emission computed tomography; NH3: ammonia; Tc: Technetium.

In summary, Precision Medicine is very important for current medicine. As in the study by Dourado et al.^1^ who found a profile of molecular response to chemotherapy treatment in a group of patients, we believe that, in the future, preventive and therapeutic approach in cardio-oncology will also be individualized. Some experimental studies have suggested that non-pharmacological approaches, like regular exercise, can be useful to prevent chemotherapy-induced cardiotoxicity, and are more precisely prescribed with the identification of patients at higher risk.^7^ Therefore, the stratification of patients and the understanding of their cellular and biochemical responses to treatments will allow a tailored approach, reducing morbidity and increasing the chances of successful treatment outcomes.
